# Supplementary material for: Spatial modelling improves genomic evaluation in Tanzanian smallholder admixed dairy cattle
Source: Genet Sel Evol. 2026 Jan 21;58:8. doi: 10.1186/s12711-025-01021-w (PMC12829002; doi:10.1186/s12711-025-01021-w)
Supplement: Supplementary file 1 — Additional_file_1: Posterior mean (left) and standard deviation (right) of the spatial effects from the GS model. [file 12711_2025_1021_MOESM1_ESM.pdf]

Additional file 1: Posterior mean (left) and standard deviation (right) of the spatial effects from the GS model

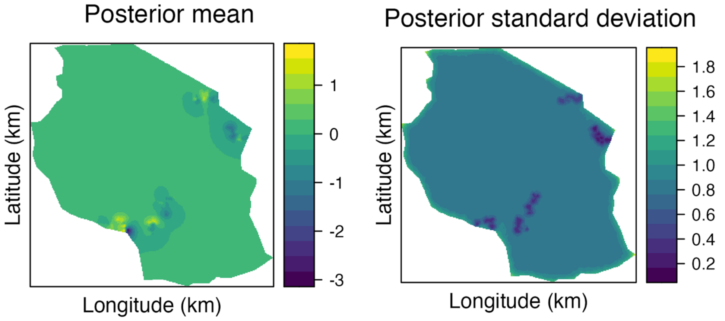

Figure S1: Posterior mean (left) and standard deviation (right) of the spatial effects (in units of phenotypic standard deviation) from the GS model
