# Supplementary material for: Spatial modelling improves genomic evaluation in Tanzanian smallholder admixed dairy cattle
Source: Genet Sel Evol. 2026 Jan 21;58:8. doi: 10.1186/s12711-025-01021-w (PMC12829002; doi:10.1186/s12711-025-01021-w)
Supplement: Supplementary file 2 — Additional_file_2: Estimates of variance components for models with permanent environmental effect. [file 12711_2025_1021_MOESM2_ESM.pdf]

Additional file 2: Estimates of variance components for models with permanent environmental effect

Table S2.1: Estimates of variance components and range by model, including permanent environment effect (posterior mean ± standard deviation)

Model	WAIC	σ^2_g	σ^2_h	σ^2_{pe}	σ^2_s	ρ (km)	σ^2_e	h^2
GP	34230	0.03±0.02	-	0.46±0.03	-	-	0.29±0.00	0.06±0.04
GPH	34192	0.02±0.00	0.36±0.02	0.12±0.01	-	-	0.29±0.00	0.03±0.01
GPS	34124	0.01±0.01	-	0.23±0.01	0.34±0.07[a]	33.5±6.6	0.29±0.00	0.02±0.01
GPHS	34130	0.01±0.01	0.13±0.01	0.11±0.01	0.33±0.06[b]	32.3±7.8	0.29±0.00	0.02±0.01

GP - model with breeding value, permanent environment effect and residual, GPH - model GP plus herd effect, GPS - model GP plus spatial effect, and GPHS - model GPH plus spatial effect;
WAIC - Watanabe–Akaike information criterion, σ^2_g - genomic variance, σ^2_h - herd variance, σ^2_{pe} - permanent environment variance, σ^2_s - "realised" spatial variance, ρ - spatial range (km), σ^2_e - Residual variance and h^2 - heritability as genomic variance divided by sum of all model variance components;
[a] - model parameter estimate was 0.57±0.14 and [b] - model parameter estimate was 0.52±0.12
