# Supplementary material for: Spatial modelling improves genomic evaluation in Tanzanian smallholder admixed dairy cattle
Source: Genet Sel Evol. 2026 Jan 21;58:8. doi: 10.1186/s12711-025-01021-w (PMC12829002; doi:10.1186/s12711-025-01021-w)
Supplement: Supplementary file 3 — Additional_file_3: Accuracy for models with permanent environmental effect. [file 12711_2025_1021_MOESM3_ESM.pdf]

Additional file 3: Accuracy for models with permanent environmental effect

Table S3.1: Cross-validation accuracy of phenotype prediction by model, percentage of exotic genome, and region

	Percentage of exotic genome				
Model	[100, 87.5] (n=731)	(87.5, 60] (n=770)	(60, 36] (n=309)	(36, 0] (n=84)	Average
GP	0.29	0.35	0.39	0.40	0.36
GPH	0.36	0.45	0.46	0.38	0.41
GPS	0.56	0.65	0.64	0.57	0.60
GPHS	0.58	0.67	0.66	0.57	0.62
	Region				
Model	NE (n=437)	NC (n=775)	SC (n=414)	SW (n=268)	Average
GP	0.08	-0.01	0.27	0.15	0.12
GPH	0.03	0.06	0.27	0.19	0.14
GPS	0.10	-0.08	0.27	0.17	0.12
GPHS	0.04	-0.03	0.16	0.13	0.08

GP - model with breeding value, permanent environmental effect and residual, GPH - model GP plus herd effect, GPS - model GP plus spatial effect, and GPHS - model GPH plus spatial effect

NE - North-East, NC - North-Central, SC - South-Central, and SW - South-West

Table S3.2: Forward validation accuracy of phenotype prediction by model, percentage of exotic genome, and region

	Percentage of exotic genome				
Model	[100, 87.5] (n=60)	(87.5, 60] (n=56)	(60, 36] (n=24)	(36, 0] (n=6)	Average
GP	0.36	0.36	0.60	0.70	0.50
GPH	0.43	0.65	0.68	0.39	0.54
GPS	0.40	0.64	0.74	0.72	0.62
GPHS	0.48	0.70	0.75	0.76	0.67
	Region				
Model	NE (n=30)	NC (n=60)	SC (n=20)	SW (n=36)	Average
GP	0.45	0.51	0.17	0.13	0.31
GPH	0.65	0.63	0.36	0.35	0.50
GPS	0.61	0.56	0.41	0.38	0.49
GPHS	0.66	0.63	0.44	0.47	0.55

GP - model with breeding value, permanent environmental effect and residual, GPH - model GP plus herd effect, GPS - model GP plus spatial effect, and GPHS - model GPH plus spatial effect

NE - North-East, NC - North-Central, SC - South-Central, and SW - South-West
